# Personalization of virtual reality for treatment of mental disorders by using a unified morphometric indicator

**DOI:** 10.1192/j.eurpsy.2022.1742

**Published:** 2022-09-01

**Authors:** U. Moskvitina

**Affiliations:** Belgorod State National Research University Institute of Medicine, Department Of Psychiatry, Narcology And Clinical Psychology, Belgorod, Russian Federation

**Keywords:** treatment of mental disorders, precision virtual reality, personalized virtual reality, a unified morphometric indicator

## Abstract

**Introduction:**

The search for approaches to creating a precision personalized virtual reality for the treatment of mental disorders is urgent. (Belkasim, 2005; Park M.J. et all., 2019).

**Objectives:**

To develop a new approach to personalize the form of virtual reality content to the neurophysiological and anatomical parameters of the patient’s brain, to improve the quality of precision VR therapy for mental disorders.

**Methods:**

The MRI study was carried out on a GE Optima 450w apparatus with a magnetic field induction of 1.5 Tesla. We used a radio frequency coil for the head. T1-weighted images were obtained with a field of view (FOV) of 24.4 x 14.8 cm and a slice thickness of 0.5 mm. Later, the images were built in three standard mutually perpendicular planes. Measurements were carried out using standard tools on an eFilm 4.0 WorkStation. FreeSurfer 4.5.0 was used to calculate the surface area of the cerebral hemispheres. To estimate the surface area of the room and the design of the shape in VR closed environment Autodesk AutoCAD 2018.

**Results:**

A new approach to personalization of the form of virtual reality content has been developed (Patent No. RU (11) 2 668 697 (13) C1, 2018;Patent No. RU (11) 2 753 234 (13) C1, 2020). It is based on the use a single morphometric indicator for a closed VR space and brain.

**Conclusions:**

Research and applied analysis of the developed approach is required. The development of this area will make it possible to create precision products for the therapy and rehabilitation of mental disorders.

**Disclosure:**

No significant relationships.

